# Erythropoiesis-stimulating agent hyporesponsiveness in chronic kidney disease: mechanistic insights and emerging precision approaches

**DOI:** 10.3389/fmed.2026.1862346

**Published:** 2026-09-22

**Authors:** Haifeng Zhang, Zihao Zhu, Xuexun Chen, Xuan Li, Jigang Shan

**Affiliations:** 1 Department of Nephrology, Affiliated Hospital of Shandong Second Medical University, Weifang, Shandong, China; 2 Department of Laboratory, The 960th Hospital of the PLA Joint Logistics Support Force, Jinan, Shandong, China

**Keywords:** anemia, CKD, EPO, erythropoietin-stimulating agent, hyporesponsiveness

## Abstract

**Background:**

Erythropoiesis-stimulating agents (ESAs) remain a cornerstone of anemia management in chronic kidney disease (CKD). However, some patients show an inadequate hemoglobin response despite appropriate or increasing ESA exposure. ESA hyporesponsiveness is clinically important because persistent dose escalation may provide limited benefit while increasing treatment burden and potential cardiovascular and thrombotic risk.

**Summary:**

ESA hyporesponsiveness is a heterogeneous phenotype driven by overlapping mechanisms, particularly inflammation, iron-restricted erythropoiesis, uremic and metabolic disturbances, and impaired erythroid responsiveness to erythropoietin. Emerging evidence further suggests that inflammation may impair erythropoiesis not only through hepcidin-mediated iron sequestration but also through disruption of EPO-responsive signaling pathways. Management should therefore prioritize systematic identification and correction of reversible causes before further intensification of erythropoietic therapy. Hypoxia-inducible factor–prolyl hydroxylase inhibitors (HIF-PHIs) may provide an alternative erythropoietic option in selected patients, but agent-specific efficacy and safety profiles differ, and evidence in rigorously defined ESA-hyporesponsive populations remains limited. Biomarker-guided and data-driven approaches may ultimately enable more mechanism-based patient stratification.

**Key messages:**

ESA hyporesponsiveness should be viewed as a multifactorial clinical phenotype rather than simply insufficient EPO replacement. Integrating mechanistic phenotyping with structured clinical evaluation may help shift anemia management from empirical ESA dose escalation toward more individualized, mechanism-based treatment.

## 1 Introduction

Chronic kidney disease (CKD) affects a substantial proportion of the global adult population (1), and anemia becomes increasingly common as kidney function declines (2). The prevalence and severity of anemia increase with advancing CKD, with a particularly high burden among patients with end-stage kidney disease (ESKD) receiving maintenance dialysis (2, 3). CKD-related anemia is associated with physical impairment, reduced quality of life, increased cardiovascular morbidity, and higher mortality (4–6).

Recombinant human erythropoietin and subsequent erythropoiesis-stimulating agents (ESAs) markedly improved the management of CKD-related anemia by raising hemoglobin (Hb) levels and reducing transfusion requirements (7–10). Nevertheless, a subset of patients exhibits an inadequate Hb response despite increasing or persistently high ESA exposure. The 2026 Kidney Disease: Improving Global Outcomes (KDIGO) guideline characterizes ESA hyporesponsiveness as failure to achieve the desired Hb response despite a substantial increase in ESA dose or as a persistent requirement for high ESA doses to maintain the intended Hb level (11). Among these individuals, further ESA dose escalation may provide limited benefit and may increase cardiovascular and thrombotic risk (11–13). ESA hyporesponsiveness should therefore be regarded not simply as insufficient erythropoietin replacement, but as a heterogeneous clinical phenotype arising from multiple interacting abnormalities in iron availability, inflammation, metabolic and endocrine homeostasis, and erythroid responsiveness (11).

Previous reviews have summarized the definitions, epidemiology, determinants, and management of ESA hyporesponsiveness in CKD (14, 15). Since their publication, however, several developments have reshaped this field. First, the 2026 KDIGO guideline provides an updated framework for the evaluation and management of persistent ESA hyporesponsiveness (11). Second, emerging mechanistic evidence suggests that inflammation may impair erythropoiesis not only through iron restriction but also through direct disruption of EPO-responsive signaling in erythroid progenitors (16). Third, accumulating clinical and regulatory evidence indicates important differences among individual HIF-PHIs in efficacy, safety, and therapeutic positioning. These developments support a shift from viewing ESA hyporesponsiveness primarily as a problem of inadequate ESA exposure toward recognizing it as a heterogeneous phenotype requiring mechanism-oriented evaluation and individualized management (17).

Accordingly, this review integrates current evidence on the mechanisms, clinical evaluation, and management of ESA hyporesponsiveness in CKD within a mechanism-oriented framework. Building on previous reviews, particular emphasis is placed on linking emerging mechanistic insights and updated clinical evidence to structured evaluation and individualized therapeutic strategies.

### 1.1 Search strategy and selection criteria

To identify relevant literature, we searched PubMed, the Cochrane Library and Embase from database inception through August 2026. Search terms included combinations of “chronic kidney disease,” “anemia,” “erythropoiesis-stimulating agents,” “ESA hyporesponsiveness,” “erythropoietin resistance,” “iron deficiency,” “hepcidin,” “inflammation,” “uremic toxins,” “secondary hyperparathyroidism,” “FGF23,” “EPO/EPOR–JAK2/STAT5,” and “HIF-prolyl hydroxylase inhibitors.” Additional targeted searches were performed for biomarkers and data-driven approaches relevant to individualized management. We prioritized recent clinical guidelines, systematic reviews, randomized controlled trials, relevant high-quality observational, and meta-analyses, and mechanistic studies. Preprints, isolated case reports, and studies without direct relevance to CKD-related anemia or ESA responsiveness were generally excluded.

## 2 Definition, epidemiology, and clinical evaluation of ESA hyporesponsiveness

### 2.1 Defining ESA hyporesponsiveness

A unified diagnostic consensus for ESA hyporesponsiveness has yet to be established, and criteria vary across guidelines and clinical studies. In general, the term describes an inadequate Hb response despite appropriate ESA therapy or a chronic reliance on elevated ESA dosages to reach or sustain target Hb concentrations. Accordingly, ESA hyporesponsiveness should be assessed longitudinally, taking into account Hb response, ESA dose trajectory, and potentially reversible causes of impaired erythropoiesis, rather than being defined by a single Hb value or ESA dose. Representative definitions proposed by major clinical guidelines are summarized in Table 1.

**Table 1:** Operational definitions of ESA hyporesponsiveness across major clinical guidelines and recommendations.

Guideline	Definition of ESA hyporesponsiveness
NKF-KDOQI, 2001 (109)	Failure to achieve the target Hb level within 4–6 months despite erythropoietin doses of 450 IU/kg/week intravenously or 300 IU/kg/week subcutaneously, or failure to maintain target Hb subsequently at that dose.
EBPG, 2004 (110)	Failure to achieve the target hemoglobin concentration despite treatment with high ESA doses, specifically more than 300 IU/kg per week of erythropoietin or 1.5 μg/kg of darbepoetin alfa, or when such high doses are required to maintain the target hemoglobin level
KDIGO, 2012 (111)	Initial hyporesponsiveness is defined as the failure of hemoglobin levels to increase from baseline after one month of treatment with an appropriate weight-based ESA dose.
	Subsequent hyporesponsiveness refers to the need for repeated ESA dose increases, typically up to 50% above a previously stable dose, to maintain target hemoglobin levels during ongoing therapy.
The UK kidney association, 2025 (112)	Failure to achieve the target Hb range despite subcutaneous erythropoietin doses exceeding 300 IU/kg/week (or intravenous doses exceeding 450 IU/kg/week), darbepoetin doses exceeding 1.5 *μ*g/kg/week, or equivalent doses of methoxy polyethylene glycol–epoetin beta, even after evaluation and treatment of other potential causes.
Japanese Society for Dialysis Therapy,2017 (113)	(1) For patients undergoing hemodialysis, failure to achieve the target Hb level despite intravenous administration of rHuEPO at 3,000 IU three times weekly (9,000 IU/week) or darbepoetin alfa at 60 μg once weekly. (2) For patients undergoing peritoneal dialysis, failure to achieve the target Hb level despite subcutaneous administration of rHuEPO at 6,000 IU once weekly (6,000 IU/week) or intravenous darbepoetin alfa at 60 *μ*g once weekly (3) For patients with non–dialysis-dependent chronic kidney disease, failure to achieve the target Hb level despite subcutaneous administration of rHuEPO at 6,000 IU once weekly (6,000 IU/week)
KDIGO 2026 (11)	Failure to achieve the desired hemoglobin response despite a substantial increase in ESA dose, or a persistent requirement for high ESA doses to maintain the intended hemoglobin level.

KDOQI, kidney disease outcomes quality initiative; NKF, national kidney foundation; Hb, hemoglobin; IU, international unit; EBPG, European best practice guideline; ESA, erythropoiesis-stimulating agent; KDIGO, kidney disease: improving global outcomes; rHuEPO, recombinant human erythropoietin.

### 2.2 Epidemiology of ESA hyporesponsiveness

The reported prevalence of ESA hyporesponsiveness varies considerably across studies because of differences in operational definitions, ESA regimens, duration of assessment, and patient populations. In contemporary dialysis cohorts, approximately 10%–15% of patients meet commonly used definitions of ESA hyporesponsiveness (18, 19). However, prevalence estimates may vary substantially when alternative definitions or different assessment periods are applied (20). Data in NDD-CKD are comparatively limited, partly because ESA use and treatment thresholds vary across disease stages and clinical settings (21). Therefore, prevalence estimates should be interpreted in the context of the definition and population studied.

### 2.3 Assessment of ESA hyporesponsiveness

Assessment of ESA responsiveness should consider both ESA dose requirements and the longitudinal Hb response. The erythropoietin resistance index (ERI) is a widely used metric of ESA responsiveness in clinical research. For patients receiving epoetin, ERI is conventionally calculated using the following equation:

$\mathrm{ERI}=\frac{weekly\;ESA\;dose\;(IU/week)}{body\;weight(kg)\times hemoglobin\;concentration(g/dL)}$. Higher ERI values indicate greater ESA requirements relative to the achieved Hb level and have been associated with higher all-cause and cardiovascular mortality in observational hemodialysis cohorts (22, 23). However, ERI is a surrogate measure rather than a direct measure of biological responsiveness to ESA therapy, because it is influenced by ESA formulation and dose, body weight, Hb variability, and the assessment period (24). ERI should therefore complement, rather than replace, longitudinal clinical assessment of ESA responsiveness.

### 2.4 Prognostic and healthcare burden of ESA hyporesponsiveness

ESA hyporesponsiveness is associated with substantial clinical burden within the CKD population. Observational studies have linked poor erythropoietic response to greater risks of cardiovascular complications, hospital admission, and mortality from any cause (9, 25–27). The observed associations should not be taken as evidence of direct causality, because inflammation, iron restriction, comorbidity burden, and higher ESA exposure may contribute to both hyporesponsiveness and adverse outcomes. ESA hyporesponsiveness is also associated with greater transfusion requirements, healthcare resource utilization, and treatment-related costs (28). Thus, poor ESA responsiveness may represent not only a therapeutic challenge but also a marker of greater underlying disease burden.

## 3 Mechanistic insights into ESA hyporesponsiveness

ESA hyporesponsiveness arises from multiple, often overlapping systemic and cellular mechanisms that limit effective erythropoiesis despite ESA exposure (Figure 1).

**Figure 1:**
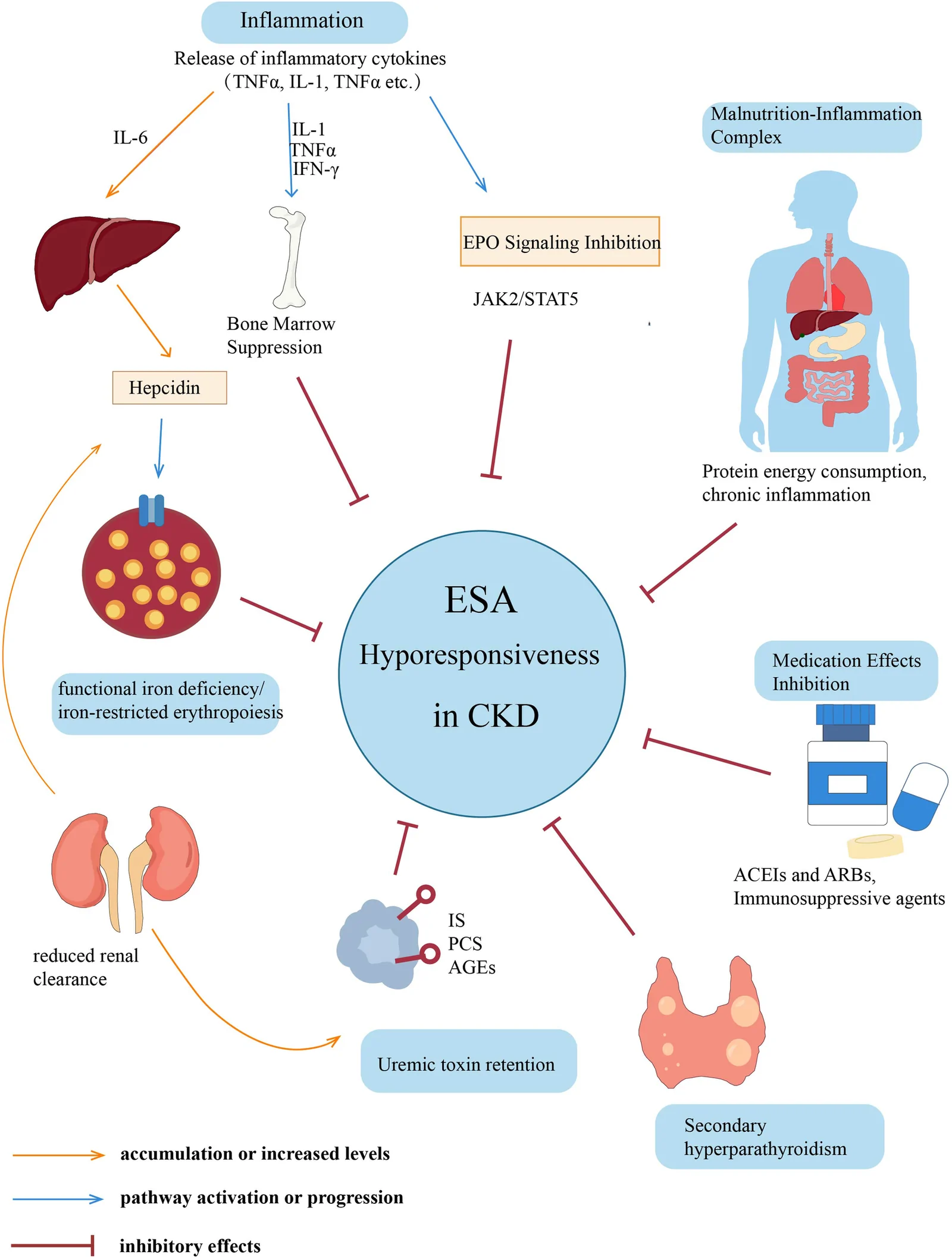
Major mechanisms contributing to erythropoiesis-stimulating agent hyporesponsiveness in chronic kidney disease. Chronic inflammation, iron-restricted erythropoiesis, impaired EPO-responsive signaling, accumulation of uremic toxins, secondary hyperparathyroidism, the malnutrition–inflammation complex, and medication-related factors may interact to reduce erythropoietic responsiveness to ESA therapy in CKD. Inflammation may contribute through both hepcidin-mediated iron restriction and suppression of erythroid progenitors and EPO/JAK2/STAT5 signaling. Blue arrows indicate pathway activation or progression, orange arrows indicate accumulation or increased levels, and red blunt-ended lines indicate inhibitory effects. AGE, advanced glycation end product; CKD, chronic kidney disease; EPO, erythropoietin; ESA, erythropoiesis-stimulating agent; IS, indoxyl sulfate; JAK2, Janus kinase 2; PCS, p-cresyl sulfate; STAT5, signal transducer and activator of transcription 5.

### 3.1 Inflammation and impaired EPO responsiveness

Chronic inflammation substantially contributes to ESA hyporesponsiveness among patients with CKD. Pro-inflammatory cytokines, including tumor necrosis factor-α (TNF-α), interleukin-1 (IL-1), and interferons, suppress erythropoiesis by inhibiting erythroid progenitor proliferation and promoting apoptosis (29–31). Inflammation may also impair cellular responsiveness to erythropoietin (EPO), providing a mechanistic link between systemic inflammatory activity and reduced effectiveness of ESA therapy.

Emerging experimental evidence suggests that high-mobility group box 1 (HMGB1), a damage-associated molecular pattern released during cellular stress and inflammation, may impair EPO-responsive signaling. Experimental work has shown that HMGB1 can restrict the expansion and survival of EPO-responsive erythroid precursors, interfere with EPO–EPOR interaction, and attenuate downstream JAK2/STAT5 and mTOR signaling (16). Suppressor of cytokine signaling (SOCS) proteins, which negatively regulate JAK/STAT signaling, may provide an additional mechanism linking persistent inflammatory activation to impaired EPO responsiveness (32–34). Importantly, however, the HMGB1–EPO signaling mechanism has been demonstrated primarily in experimental models of anemia of inflammation rather than CKD-specific ESA hyporesponsiveness. Its contribution to ESA resistance in CKD therefore remains to be established. Consistent with a broader role of inflammation, elevated inflammatory markers have been associated with greater ESA requirements and reduced ESA responsiveness in dialysis populations (35–37).

### 3.2 Iron dysregulation and hepcidin excess

Impaired iron availability is a major contributor to ESA hyporesponsiveness in CKD, arising from either absolute iron deficiency or iron-restricted erythropoiesis. Whereas absolute iron deficiency reflects depletion of body iron stores, iron-restricted erythropoiesis is characterized by inadequate iron delivery to erythroid precursors despite preserved or increased iron stores and is particularly relevant in the inflammatory milieu of CKD (11, 38). Hepcidin is a central mediator linking inflammation to iron-restricted erythropoiesis. In CKD, inflammation, particularly activation of the IL-6/STAT3 pathway, enhances hepatic hepcidin synthesis, while impaired renal clearance may further contribute to elevated circulating hepcidin levels (38–41). Hepcidin binds to ferroportin and promotes its internalization and degradation (42), ultimately restricting both intestinal iron uptake and iron mobilization from macrophages. Consequently, iron becomes sequestered and less accessible for erythropoiesis, potentially attenuating the therapeutic effect of ESAs even when overall iron stores appear adequate (39–41) (Figure 2). Consistent with this mechanism, higher circulating hepcidin concentrations have been associated with greater ESA requirements and higher erythropoietin resistance index values in maintenance hemodialysis populations (43, 44). Thus, the hepcidin–ferroportin axis provides a key mechanistic link between inflammation, iron-restricted erythropoiesis, and ESA hyporesponsiveness in CKD.

**Figure 2:**
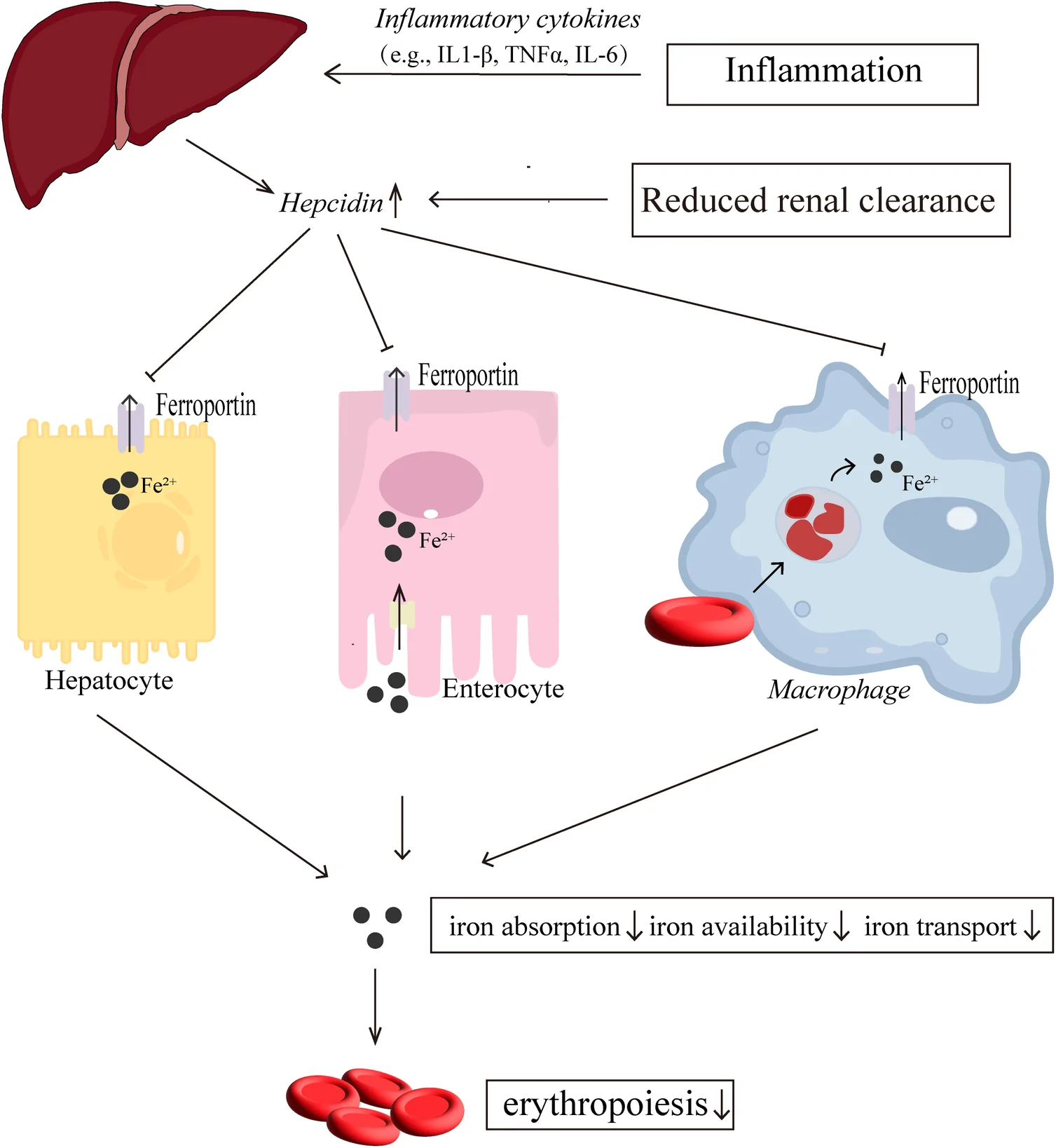
Inflammation and reduced renal clearance contribute to elevated circulating hepcidin levels, thereby reducing intestinal iron absorption and iron release from macrophages and limiting iron availability for bone marrow erythropoiesis.

### 3.3 Uremic toxins and bone marrow dysfunction

Uremic toxin accumulation in advanced CKD may further suppress erythropoiesis. Experimental studies suggest that protein-bound solutes, particularly indoxyl sulfate, can inhibit erythroid progenitor proliferation and differentiation and promote apoptosis and cellular senescence (45, 46). Other retained solutes, including advanced glycation end products and *p*-cresyl sulfate, may amplify oxidative stress and inflammatory signaling (47–49). However, direct evidence linking individual uremic toxins to reduced responsiveness to exogenous ESA therapy remains limited. Their contribution to ESA hyporesponsiveness may therefore be mediated largely through inflammation, oxidative stress, and bone marrow dysfunction.

### 3.4 Metabolic and endocrine disturbances

Secondary hyperparathyroidism (SHPT) is a potentially reversible contributor to reduced ESA responsiveness in advanced CKD. Elevated parathyroid hormone levels may impair erythropoiesis through shortened erythrocyte survival and bone marrow fibrosis (50, 51), while severe SHPT has been associated with greater ESA requirements in clinical studies (51–53).

Fibroblast growth factor-23 (FGF23) has also been implicated as a possible link between disordered mineral metabolism and impaired erythropoiesis. Experimental studies suggest that excess FGF23 can suppress erythropoiesis (54, 55), and recent observational data in hemodialysis patients have associated higher circulating FGF23 levels with greater ESA requirements; higher erythropoietin resistance index values were also observed for some FGF23 measures (56). However, whether FGF23 directly contributes to ESA hyporesponsiveness in humans remains uncertain, and its clinical significance requires further investigation.

### 3.5 Malnutrition and micronutrient deficiency

Protein-energy wasting may contribute to impaired ESA responsiveness in advanced CKD, often in close association with chronic inflammation. Lower serum albumin and adverse nutritional–inflammatory status have been associated with reduced erythropoietic responsiveness in dialysis populations (57, 58). However, these associations likely reflect overlapping effects of nutritional depletion, inflammation, and comorbidity rather than isolated nutritional deficiency alone (59).

Folate or vitamin B12 deficiency may impair erythroid DNA synthesis and red blood cell maturation and represent potentially reversible contributors to an inadequate ESA response (11, 60–62).

### 3.6 Medication-related contributors to ESA hyporesponsiveness

Several medications and treatment-related factors may contribute to impaired ESA responsiveness. Renin–angiotensin system inhibitors, particularly at higher doses, have been associated with modest reductions in erythropoietic response and greater ESA requirements in some dialysis studies (63, 64). These observations should not, however, be interpreted as a reason to discontinue clinically indicated renin–angiotensin system blockade given its established cardiorenal benefits. Mycophenolate mofetil and mTOR inhibitors may suppress erythropoiesis, particularly in kidney transplant recipients (65, 66), and mTOR inhibition has also been associated with increased erythropoietin resistance (67). Aluminum overload is a well-established historical cause of ESA resistance in dialysis patients (68, 69), although clinically significant aluminum toxicity is now uncommon. Medication exposure and other treatment-related factors should therefore be reviewed as possible contributors when ESA responsiveness is unexpectedly poor, while changes in clinically indicated therapies should be individualized.

Overall, ESA hyporesponsiveness reflects the interaction of multiple systemic and cellular abnormalities rather than a single mechanism. Translating these mechanisms into clinical practice requires a structured evaluation to identify the dominant and potentially reversible contributors in individual patients (Figure 3).

**Figure 3:**
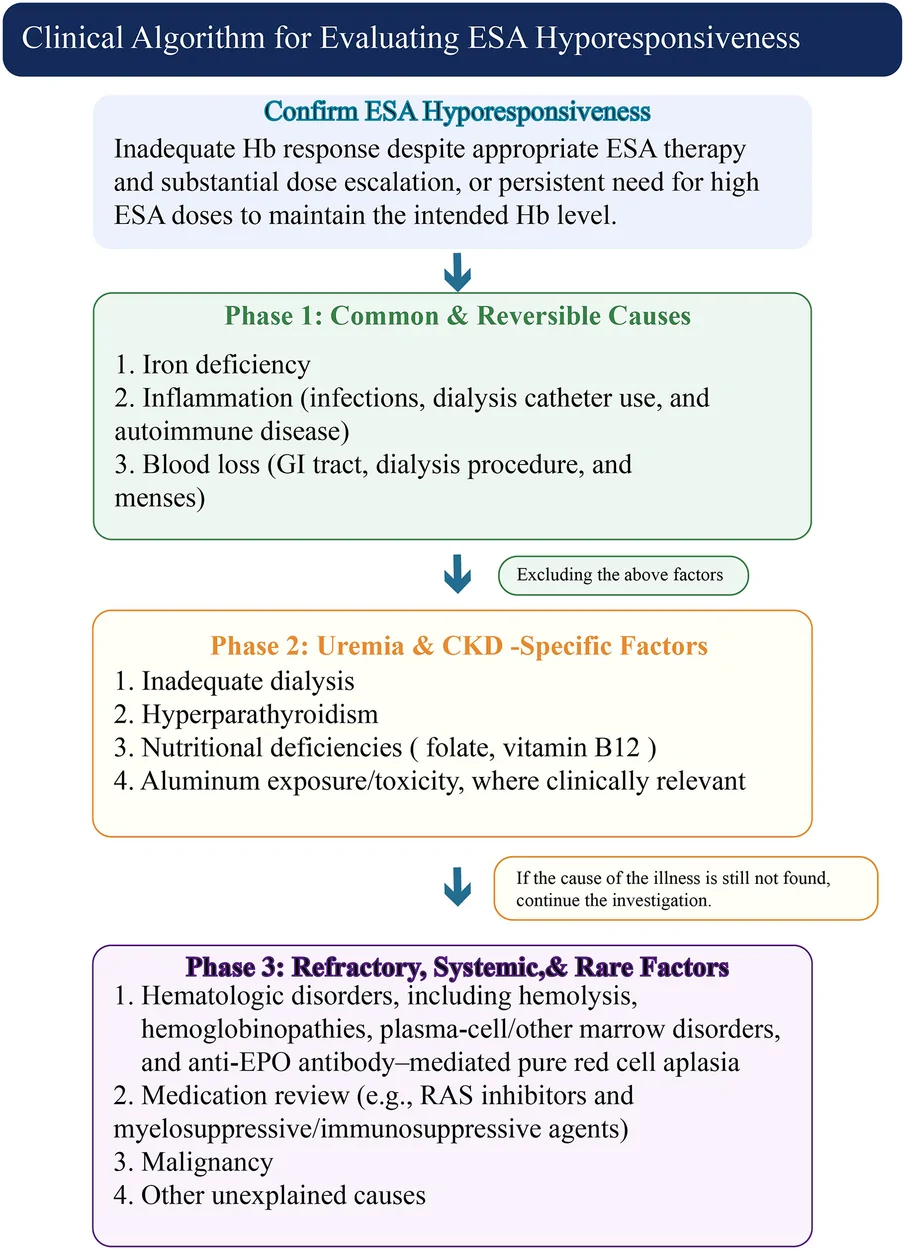
Clinical algorithm for evaluating ESA hyporesponsiveness in chronic kidney disease. Evaluation should proceed from common and potentially reversible causes to CKD- and dialysis-related contributors and, when hyporesponsiveness persists, to systemic, hematologic, medication-related, malignant, and rare causes. ESA, erythropoiesis-stimulating agent; CKD, chronic kidney disease; EPO, erythropoietin.

## 4 Conventional management strategies for ESA hyporesponsiveness

### 4.1 Iron optimization

Iron deficiency and iron-restricted erythropoiesis should be identified and corrected before poor Hb response is attributed to intrinsic ESA hyporesponsiveness (11). Although oral iron may be appropriate in selected patients, its effectiveness can be limited by gastrointestinal intolerance and impaired absorption, particularly in the presence of inflammation (70). Among patients receiving hemodialysis, intravenous (IV) iron therapy generally achieves more effective iron repletion and can reduce ESA requirements (11, 71). The PIVOTAL trial demonstrated that a proactive high-dose IV iron strategy, compared with a reactive low-dose strategy, reduced ESA requirements and was associated with improved clinical outcomes in patients receiving maintenance hemodialysis (71). Although meta-analytic evidence has raised concerns regarding a potential association between IV iron administration and infection risk (72), a prespecified secondary analysis of PIVOTAL found no increase in infection risk with the proactive high-dose strategy compared with the reactive low-dose strategy (73).

Other iron-containing therapies, such as ferric citrate, may also reduce ESA requirements, likely through improved iron availability. Improvements in phosphate control and reductions in FGF23 have also been reported, although their independent contribution to reduced ESA requirements remains uncertain; current evidence does not establish a direct enhancement of ESA action by ferric citrate (74, 75). Overall, optimization of iron availability remains a fundamental component of the evaluation and management of ESA hyporesponsiveness (11).

### 4.2 Inflammation control and infection management

Identification and treatment of underlying infectious or inflammatory conditions are important components of the management of ESA hyporesponsiveness. In patients receiving hemodialysis, occult infection and dialysis-related inflammatory stimuli may sustain systemic inflammation and contribute to increased ESA requirements (76–78). Accordingly, potentially reversible sources of inflammation should be actively investigated before ESA dose escalation.

Evidence supporting pharmacological anti-inflammatory therapy specifically for ESA hyporesponsiveness remains limited. Among targeted approaches, IL-6 pathway inhibition with ziltivekimab has been associated with improvements in Hb and iron-related parameters in patients with CKD (79). In a small randomized phase 1/2 study of patients receiving hemodialysis with systemic inflammation and ESA hyporesponsiveness, ziltivekimab reduced ESA requirements and erythropoietin resistance index values (80). By contrast, the HERO trial did not demonstrate a significant improvement in ESA responsiveness with pentoxifylline (81). These findings support inflammation as a potentially modifiable driver of poor ESA response, but targeted anti-inflammatory therapy should currently be considered investigational rather than routine treatment.

### 4.3 Correction of secondary hyperparathyroidism and nutritional deficiencies

Secondary hyperparathyroidism (SHPT) and nutritional abnormalities should be considered potentially reversible contributors to poor ESA responsiveness. In patients with severe SHPT, treatment directed at controlling parathyroid hormone levels may improve anemia management. Cinacalcet has been associated with reduced ESA requirements and lower ERI values in dialysis patients with SHPT (82), while parathyroidectomy has also been associated with reduced ESA requirements in patients with medically refractory SHPT (83, 84). However, these interventions should primarily be guided by established indications for CKD–mineral and bone disorder rather than used solely to improve ESA responsiveness.

Protein-energy wasting and other nutritional abnormalities have been associated with reduced ESA responsiveness in maintenance dialysis populations (85, 86). Nutritional assessment should therefore form part of the evaluation of persistent hyporesponsiveness, with correction of documented deficiencies, including folate and vitamin B12 deficiency, when present (11, 87). Evidence that nutritional interventions alone substantially reverse established ESA hyporesponsiveness, however, remains limited.

### 4.4 Optimization of dialysis adequacy

Inadequate dialysis is a potentially reversible contributor to poor anemia control and should be assessed in individuals demonstrating sustained ESA hyporesponsiveness (88). Ensuring an adequate dialysis dose and addressing problems that compromise dialysis delivery therefore remain fundamental components of management.

Enhanced removal of middle-molecular-weight uremic toxins through alternative blood purification strategies may improve selected indices of ESA responsiveness. Randomized and comparative studies have reported reductions in ESA requirements or erythropoietin resistance index values with medium cut-off dialyzers or high-volume online hemodiafiltration compared with conventional hemodialysis (89–91). However, the magnitude and consistency of these effects vary across studies. Current evidence does not support changing dialysis modality solely to improve ESA responsiveness. Such approaches should therefore complement, rather than replace, correction of established reversible causes of hyporesponsiveness.

Overall, management of ESA hyporesponsiveness should begin with systematic identification and correction of potentially reversible causes rather than empirical ESA dose escalation. Iron restriction, inflammation or infection, metabolic and nutritional abnormalities, and inadequate dialysis should be addressed before alternative erythropoietic strategies are considered (11) (Figure 4).

**Figure 4:**
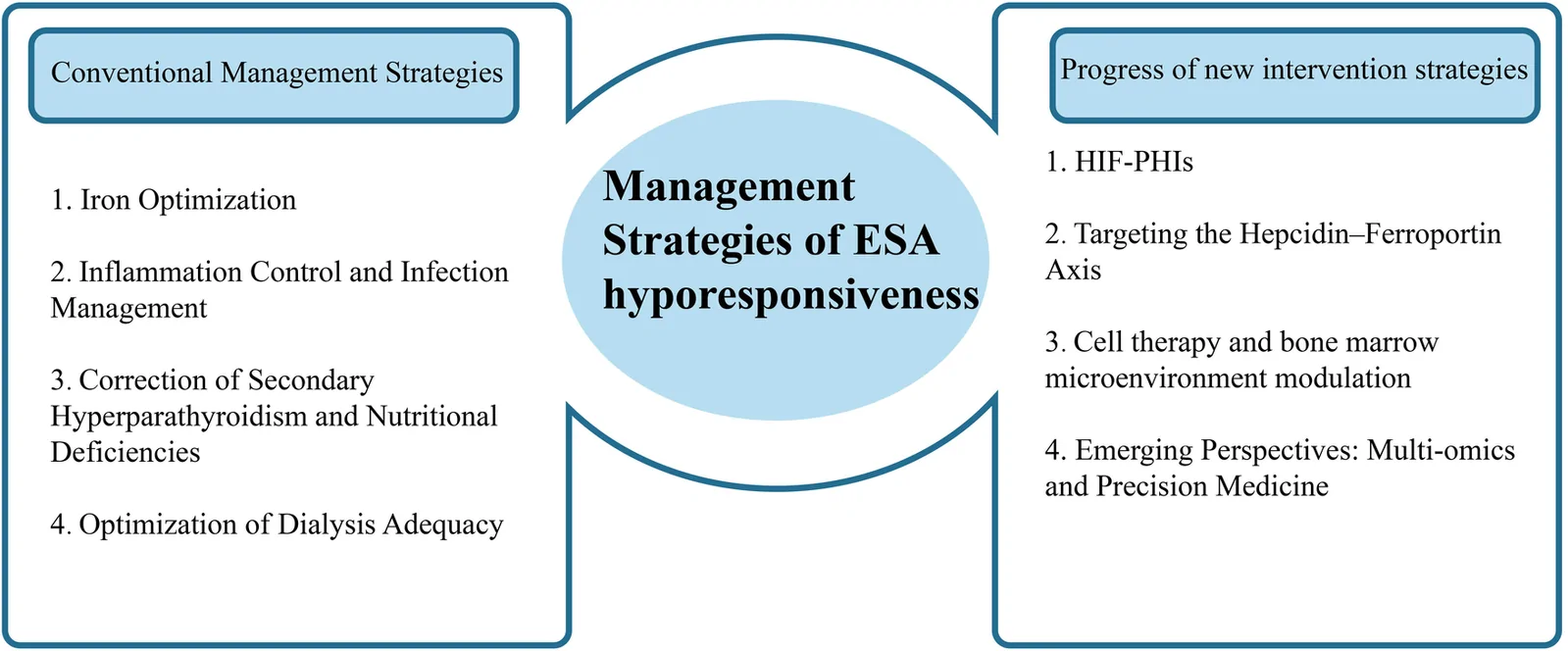
Management strategies for ESA hyporesponsiveness.

## 5 Emerging intervention strategies

### 5.1 HIF-PHIs as a potential therapeutic strategy for ESA hyporesponsiveness

HIF-PHIs stabilize hypoxia-inducible factors, thereby increasing endogenous erythropoietin production and promoting iron availability through effects on hepcidin and other iron-handling pathways (92–94). These effects provide a mechanistic rationale for considering HIF-PHIs in selected patients with poor ESA responsiveness, particularly when inflammation and iron restriction are prominent. Nevertheless, clinical evidence specifically derived from rigorously defined ESA-hyporesponsive populations remains limited (11). Importantly, individual HIF-PHIs should not be regarded as interchangeable, because their efficacy, cardiovascular safety profiles, clinical evidence, and regulatory status differ across agents and patient populations. In ASCEND-D, daprodustat was noninferior to conventional ESAs with respect to the change in Hb from baseline and cardiovascular outcomes in patients with dialysis-dependent CKD (95). Similarly, in the INNO₂VATE trials, vadadustat was noninferior to darbepoetin alfa with respect to cardiovascular safety and the correction and maintenance of Hb concentrations in patients with dialysis-dependent CKD (96). By contrast, in the PRO₂TECT trials involving patients with nondialysis-dependent CKD, vadadustat met the prespecified noninferiority criterion for hematologic efficacy but not for cardiovascular safety compared with darbepoetin alfa (97). For roxadustat, pooled analyses suggest that the Hb response may be less attenuated by baseline inflammation (98), and small exploratory studies have reported improved anemia parameters after switching ESA-hyporesponsive hemodialysis patients to roxadustat (99). Nevertheless, these observations do not establish superiority over ESAs in ESA-hyporesponsive patients, and dedicated randomized trials remain scarce. The 2026 KDIGO guideline recommends ESAs rather than HIF-PHIs as first-line erythropoietic therapy, but allows a trial of HIF-PHI in selected patients with ESA hyporesponsiveness after discussion of benefits and potential risks. ESA and HIF-PHI therapy should not be combined; the lowest effective HIF-PHI dose should be used, and treatment should be discontinued if an adequate erythropoietic response is not achieved within 3–4 months (11). Given cardiovascular and thrombotic risks and unresolved concerns regarding malignancy, HIF-PHIs should not be used in patients with active malignancy or a recent cardiovascular or vascular thrombotic event (11). Regulatory approval and labeled indications vary across agents and regions and should therefore be considered when selecting an individual HIF-PHI.

### 5.2 Targeting the hepcidin–ferroportin axis

The hepcidin–ferroportin axis represents a mechanistically attractive therapeutic target for iron-restricted erythropoiesis in CKD. Several hepcidin-neutralizing or iron-mobilizing approaches have entered early clinical development. In a phase I study, the hepcidin antagonist PRS-080 increased circulating iron concentrations and transferrin saturation in patients receiving maintenance hemodialysis, but did not produce consistent improvements in Hb (100). Other strategies targeting hepcidin or ferroportin have shown expected pharmacodynamic effects, but clinical evidence in CKD remains limited (101, 102). Importantly, whether these approaches can achieve sustained Hb improvement or reduce ESA requirements in patients with established ESA hyporesponsiveness remains unknown.

Taken together, emerging therapeutic approaches to ESA hyporesponsiveness extend beyond conventional ESA dose escalation and include HIF-PHIs and experimental strategies targeting iron regulation and inflammation. However, evidence remains limited in patients with rigorously defined ESA hyporesponsiveness, and most studies have focused on hematologic rather than patient-centered outcomes. Further dedicated trials are required to establish efficacy, safety, and appropriate therapeutic positioning.

## 6 Future directions and challenges

A major challenge in ESA hyporesponsiveness is its biological heterogeneity. Similar clinical phenotypes may arise from different combinations of inflammation, iron restriction, metabolic and endocrine abnormalities, uremic toxicity, and impaired erythroid responsiveness. Future studies should therefore move beyond treating ESA hyporesponsiveness as a single entity and define biologically distinct phenotypes that may respond differently to mechanism-based interventions. In particular, further investigation is needed to determine whether disruption of EPO-responsive signaling represents a clinically relevant mechanism in specific subsets of patients with CKD.

Future clinical trials should specifically enroll patients with clearly defined ESA hyporesponsiveness rather than extrapolating efficacy from general CKD anemia populations. Standardized definitions of hyporesponsiveness, documentation of reversible causes, and stratification according to major biological drivers will be important for improving comparability across studies. In addition to Hb response and ESA dose reduction, clinically meaningful outcomes—including cardiovascular and thrombotic events, transfusion requirements, hospitalization, quality of life, and mortality—should be incorporated into trial design.

Emerging research on the bone marrow microenvironment may provide additional mechanistic and therapeutic insights into ESA hyporesponsiveness. Erythroblastic island macrophages and other components of the marrow niche regulate erythroid maturation and EPO responsiveness (103, 104). Cell-based and extracellular vesicle approaches have also shown erythropoietic effects in preclinical CKD models; for example, EPO-expressing mesenchymal stem cell-derived extracellular vesicles improved anemia in a CKD mouse model (105). However, these strategies remain preclinical, and there is currently no evidence that they improve ESA responsiveness in patients with CKD.

Improved patient stratification may ultimately facilitate more individualized treatment. Biomarker panels integrating indices of inflammation, iron metabolism, nutritional status, and erythropoietic activity may help identify dominant mechanisms of hyporesponsiveness, while multi-omics approaches could further refine mechanistic phenotyping. Data-driven decision-support tools have also shown potential to optimize anemia management and ESA use in hemodialysis populations (106), but prospective validation and demonstration of generalizability are required before such approaches can be incorporated into routine management of ESA hyporesponsiveness.

Although this review focuses on CKD, the role of anemia-directed therapies in acute kidney injury (AKI) and acute kidney disease (AKD) also warrants consideration. However, direct therapeutic evidence remains limited and is derived predominantly from AKI populations. In the EAKI multicenter randomized pragmatic trial, recombinant human erythropoietin did not reduce red blood cell transfusion requirements or improve renal recovery or survival in patients with AKI and anemia (107). More recently, a phase 2 randomized trial found that a single intravenous iron dextran infusion in patients with AKI and iron deficiency did not improve 90-day major adverse kidney events or Hb concentrations compared with standard care (108). These findings do not support direct extrapolation of CKD-based ESA or iron strategies to AKI/AKD, and dedicated studies, particularly in AKD, remain needed.

## 7 Conclusions

ESA hyporesponsiveness in CKD is a heterogeneous clinical phenotype arising from multiple interacting abnormalities, including iron restriction, chronic inflammation, metabolic and endocrine disturbances, uremic factors, and altered erythroid responsiveness to EPO. Management should therefore prioritize systematic identification and correction of potentially reversible causes rather than empirical ESA dose escalation. HIF-PHIs may provide an alternative erythropoietic option in selected patients, but evidence specifically derived from rigorously defined ESA-hyporesponsive populations remains limited. Other mechanism-based approaches, including therapies targeting the hepcidin–ferroportin axis, remain investigational. Further progress will require standardized definitions, mechanistic phenotyping, and dedicated clinical trials incorporating both hematologic and patient-centered outcomes. A mechanism-oriented approach may ultimately enable more individualized and effective management of ESA hyporesponsiveness in CKD.
